# Detection of *Toxoplasma gondii* copro-prevalence by polymerase chain reaction using repetitive 529 bp gene in feces of pet cats (*Felis catus*) in Yogyakarta, Indonesia

**DOI:** 10.14202/vetworld.2018.1338-1343

**Published:** 2018-09-28

**Authors:** Muhammad Hanafiah, Joko Prastowo, Sri Hartati, Dwinna Aliza, Raden Wisnu Nurcahyo

**Affiliations:** 1 Parasitology Laboratory, Faculty of Veterinary Medicine, Syiah Kuala University, Banda Aceh, Indonesia; 2 Department of Parasitology, Faculty of Veterinary Medicine, Gadjah Mada University, Yogyakarta, Indonesia; 3 Department of Clinic, Faculty of Veterinary Medicine, Gadjah Mada University, Yogyakarta, Indonesia; 4 Pathology Laboratory, Faculty of Veterinary Medicine, Syiah Kuala University, Banda Aceh, Indonesia

**Keywords:** copro-prevalence, pet cat, polymerase chain reaction, *Toxoplasma gondii*

## Abstract

**Aim:**

The aim of this research was to determine the copro-prevalence of *Toxoplasma gondii* using polymerase chain reaction (PCR) with repetitive 529 bp gene and to construct the phylogenetic tree of *Toxoplasma* oocyst from pet cats in Yogyakarta.

**Materials and Methods:**

9 of 132 pet cat samples which serologically positive for *Toxoplasma* were used in this research. To determine the copro-prevalence of *T. gondii* in pet cat, 10 g of feces samples taken from practitioners and household cats in Yogyakarta were used in the PCR method utilizing repetitive 529 bp gene sequences.

**Results:**

The result shows that copro-prevalence by PCR using repetitive 529 bp gene was 33.3% (3/9). The phylogenetic tree of *Toxoplasma* grouped into two clades, which clade 1 consists of *Toxoplasma* isolates collected from pet cats in Yogyakarta Indonesia and *T. gondii* isolates from China and in clade 2 consist of the *T. gondii* isolates from India.

**Conclusion:**

Copro-prevalence of *T. gondii* in pet cats in Yogyakarta by means of PCR using repetitive 529 bp gene is around 33.3%.

## Introduction

*Toxoplasma gondii* infections are prevalent in humans and animals worldwide. It has been estimated that one-third of the world population has been exposed to this parasite [1]. Toxoplasmosis showed no specific symptoms on domestic cats but may cause chronic illness and clinical symptoms in neonates, geriatric, and immunocompromised animals [2]. Felines are the final or definitive hosts of *T. gondii* while human and all other warm-blooded animals are as intermediate hosts [3]. In humans, the most common routes of *T. gondii* transmission are through ingestion of undercooked meat which contains cysts, poorly washed vegetables, and water or soil contaminated with oocysts [4]. Determination of toxoplasmosis diagnosis is inaccurate by clinical approach since infection is asymptomatic or subclinical in chronic infection, especially on immunocompetent hosts. Some clinical symptom data have been collected from the examination of body temperature, breath, and pulse frequencies [5]. The results showed that all samples still in the normal standard of healthy cat. However, the clinical data were not specific enough to diagnose cat with toxoplasmosis [6]. Various serological and molecular tests have been widely used by researchers in epidemiological studies on animal and human toxoplasmosis worldwide [7].

Molecular diagnostic method for toxoplasmosis such as polymerase chain reaction (PCR) which is simple and sensitive has been produced and able to be implemented on all clinical samples [8-10]. Diagnosis of toxoplasmosis has been improved by the emergence of molecular technologies to amplify parasite nucleic acids. Among these, PCR-based molecular techniques have been used for the genetic characterization of *T. gondii* [11]. PCR requires only common molecular biology experience since it easily differentiates *T. gondii* from other cyst forming eukaryotes and is highly sensitive [12,13].

Molecular diagnostics of toxoplasmosis were generally based on detection of specific DNA sequences. Some use B1 gene that has 35 copies in the genome, but others use DNA R529 bp fragment that has 200-300 copies in genome, internal transcribed spacer - 1 that consists 110 gen copies, or 18s rRNA gene sequence [4]. Qualitative PCR to detect single gen copy such as P30 was not sensitive and less commonly used for the diagnostic purpose [14]. Garcia *et al*. [15] have used repetitive 529 bp sequence and Tox4 and Tox5 primers to detect *T. gondii* within pig tissue and compared it with mouse bioassay and histopathology. The advance of molecular-based copro-diagnostic method is hoped to be used in detecting *T. gondii* [16-18].

The objective of this research was to determine the copro-prevalence of *T. gondii* using PCR with repetitive 529 bp gene and to construct *Toxoplasma* oocyst phylogenetic tree from pet cats in Yogyakarta.

## Materials and Methods

### Ethical approval

This study was approved by the Ethics Committee of Ethical Clearance for Pre-Clinical Research, Integrated Research and Testing Laboratory, Gadjah Mada University, Yogyakarta, Indonesia (Approval no. 00111/09).

### Sample preparation

Samples used in this research were 132 cats obtained from some areas which randomly selected in Yogyakarta, namely Yogyakarta city, Sleman, Bantul, Kulon Progo, and Gunung Kidul. 10 g of feces of each cat was collected and put into plastic bag and labeled before brought to the Laboratory of Parasitology Faculty of Veterinary Medicine Gajah Mada University. All samples were stored in 4°C refrigerator.

### Centrifuge method

2 g of feces samples were placed in a mortar, added with distilled water, stirred until mixed, and then poured into centrifuge tube up to ¾ of the tube’s height. The tube was then spun at 2000 rpm for 5 min. The clear supernatant was discarded, and saturated NaCl solution was added until ¾ of tube’s height after which the tube was spun again in 2000 rpm for 5 min. The centrifuge tube was placed straight up in a rack and then saturated NaCl was dripped until the water surface reached the top and seemed concave before stored for 3 min. Object glass was touched on the water surface carefully and reversed. The surface of the object glass which touched the water surface was covered by cover glass and examined under the microscope [19].

### *Toxoplasma* oocyst DNA isolation

Feces samples obtained from feces examination by floatation method were carefully transferred to a new tube, where distilled water was added up to 1.5 of the tube’s height. Samples were then centrifuged in 2000 g for 15 min. The oocyst was then extracted by 200 ul of ASL from the QIAamp DNA Stool Mini Kit (QIAGEN). Extraction was done by adding proteinase in 60°C temperature for 1 h. The solution was then eluted within QIAAmp column up until 200 µl. DNA samples were then placed according to QIAAmp kit direction in −20°C until used.

### *T. gondii* specific PCR

PCR was carried out with a total volume of 50 ml solution consisting of 10 ml of sample DNA. Primer TOX4 (50-CGCTGCAGGG AGGAAGACG AAAGTTG-30) and TOX5 (50-CGCTGCAGAC ACAGTGCATCTGGATT-30) used 5’ and 3’ end in 529 bp repetitive sequence [20]. PCR mixture containing 0.2 mM of each primers, 100 mM dNTP (Fermentas), 60 mM Tris-HCl (pH 9.0), 15 mM (NH_4_) 2SO4, 2 mM MgCl_2_, and 1U Biotaq (Bioline, MA, USA) per reaction. Amplification was done by PTC-150 MiniCycler thermocycler (MJ Research Inc, MA, USA) with the first denaturation for 7 min in 94°C, followed by 35 cycles of 1 min in 95°C, 1 min in 60°C, 1 min in 72°C, and final incubation of 10 min in 72°C. Subsequently, the positive control that was DNA comparative to five *T. gondii* tachyzoites and negative control without DNA product were run in 1.1% agarose gel electrophoresis added with ethidium bromide, using 1Kb DNA ladder as a marker (Biolabs, MA, USA).

### Sequencing

PCR product obtained was sequenced by ABI Prism BigDye Terminator Cycle Sequencing Kit (Applied Biosystems, Foster City, CA, USA). Sequence product was analyzed by program *BioEdit* and MEGA 6 software (USA).

### Statistical analysis

*T. gondii* copro-prevalence data in pet cats were analyzed descriptively. The *Toxoplasma* oocyst was then constructed for the phylogenetic tree using the neighbor-joining method.

## Results

### Centrifuge method

**Figure-1:**
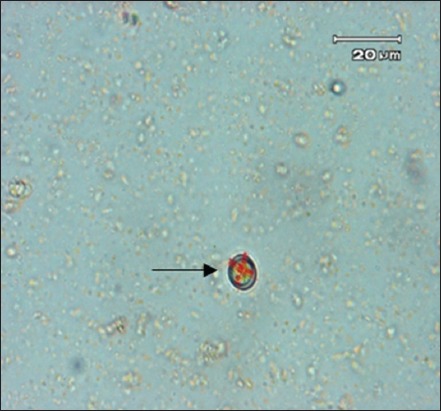
Micrograph of *Toxoplasma gondii* oocyst sheds by naturally infected pet cat (scale bar=20 µm) (arrow).

The examination using the centrifugation method showed that 9 out of 132 pet cats were positive toxoplasmosis proven by the presence of *Toxoplasma* oocyst in pet cat feces samples in Yogyakarta appeared as shown in Figure-1.

The centrifuged method revealed that the *Toxoplasma* oocyst was found in pet cat feces, proven by the size of the oocyst with the diameter ranging from 9.37 µm×11.25 µm. The finding of the measurement indicates that the oocyst was positive belongs to toxoplasma genus not from other protozoa such as *Hammondia* spp. or *Sarcocystis* spp.

### Molecular identification and sequencing analysis

**Figure-2:**
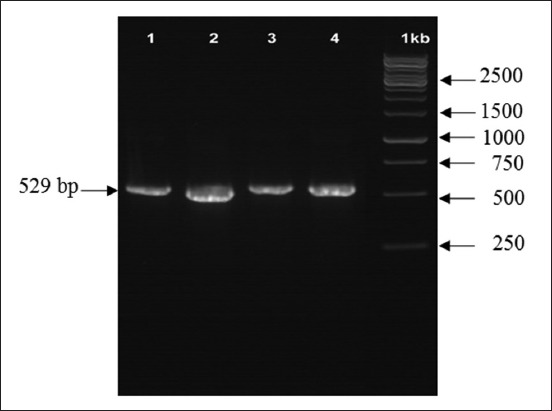
Amplification result of repetitive 529 gene *Toxoplasma* oocyst on 1% agarose gel. Annotation: M: 1 kb DNA ladder, 1-3: Samples, 4: Control (tachyzoite).

**Figure-3:**
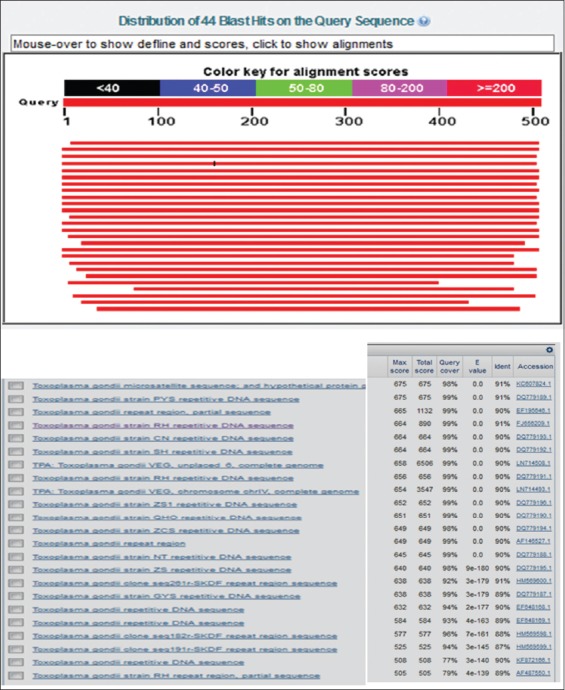
The result of output BLASTN sequence repetitive R529 (sequencing product).

Partial amplification of repetitive 529 bp gene showed that 3 of 9 samples (33.3%) were positive as shown in Figure-2. BLASTN (Basic Alignment Search Tool) analysis was conducted online using http//www.ncbi.nlm.nih.gov. The analysis results are shown in Figure-3.

Figure-3 shows the alignment result, which revealed that the homology level of *T. gondii* repetitive sequencing products compare to other strain is varies ranging from 83% to 91%. The identical level of 91% are revealed from sequence in Genbank accession number of KC607824.1, DQ779189.1, FJ656209.1 and HM569600.1, while the identical level of 90% was with EF195646.1, DQ779193.1, DQ779192.1, LN714508.1, DQ779191.1, LN714493.1, DQ779190.1, DQ779194.1, AF146527.1, DQ779188.1, DQ779195.1, KF872166.1, and EF648168.1, identical level of 89% with DQ779187.1, EF648169.1, and AF487550.1, identical level of 88% with HM569598.1, share identical level of 87% with HM569599.1, identical level of 84% with HM569602.1, identical level of 83% with HM569597.1 and HM569603.1.

**Figure-4:**
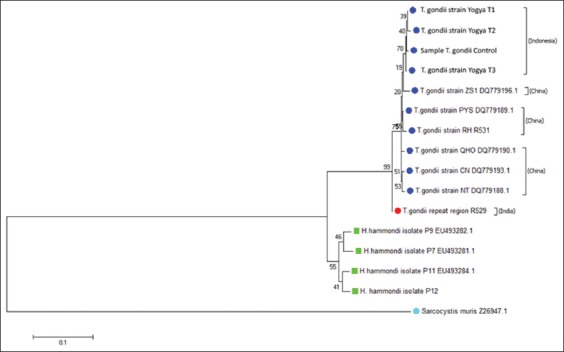
Phylogenetic tree analysis based on repetitive 529 DNA partial sequence that used for confirmative identification of *Toxoplasma gondii* isolates by neighbor-joining method.

The genetic relatedness of the identified strain was determined through phylogenetic analysis (Figure-4).

## Discussion

### Centrifuge method

The examination using the centrifugation method shows that 11 of 132 cats were positive toxoplasmosis proven by the presence of *Toxoplasma* oocyst (Figure-1). Oocyst of *T. gondii* is structurally similar to those of *H. hammondia* and *Sarcocystis* sp. that is also using cats as definitive host. Simamora *et al*. [21] suggested some further procedures to identify *T. gondii* oocysts in feline feces which were used in this study: (i) Measurement of oocyst diameter, (ii) detection of *T. gondii* specific 529 bp amplicon, (iii) recovery of *T. gondii* cysts from mice and cat tissue, and (iv) infectivity of cysts to mice. Based on the measurement, it showed that the size of the oocyst found was ranging from 9.37 µm×11.25 µm, which in range of the *Toxoplasma* oocyst (<12 µm) [21].

The copro-prevalence level of *Toxoplasma* in cat in this study was 8.33% which higher than the study reported by Liang *et al*. [22] which was 4% in Kunming, China. However, this result was lower than those reported by Dubey *et al*. [23] which was 19% in Ethiopia and in wildcat (90%) and pet cat (36%) in Tehran [24]. In addition, the level infection of *T. gondii* in cat in Kerman was 32.1% [25]. These various results showed not only depend on the exposure of oocyst but also the sensitivity of the method used by researcher.

### Molecular identification and sequencing analysis

The observation of pet cat oocyst in Yogyakarta using PCR confirmation based on repetitive 529 bp gene (Figure-2) obtained the copro-prevalence level was 33.3%. This is higher than those reported by Dubey [26] that was 9.0%. Virgen *et al*. [27] and Bolais *et al*. [28] stated that in a study using PCR confirmation in wildcat found that the prevalence was 5.9% and 3.74% whereas [29] using nested PCR in South Korea found higher prevalence (47.2%) in cat.

The overall prevalence of seroreagent cats from the Brazilian semi-arid for *T. gondii* was 43.8%. We found a prevalence of 47.7% in domiciled cats and 36.2% in stray cats [30]. The incidence of oocyst shedding in the cat population studied was significantly higher than expected and higher than found in most cat population worldwide [31]. Three samples, (from 1 stray and 2 indoor/outdoor pets), yielded sequences with high identity to *T. gondii* isolates, and were identified as positive for *T. gondii* oocytes.

The study conducted by Bizhga [31] demonstrated that the prevalence of *T. gondii* infection among pet cats was low due to the cats being strictly kept indoors, restricted from eating raw food and uncooked meat and having no chance to contact with other wild animals and the ground after adoption. The positive result of *T. gondii* in pet cats was obtained using ELISA while in stray cats from the animal shelter and clinics revealed the positive result of *T. gondii* by using PCR. Furthermore, the examination of 442 pet cats which always contact with human resulted in 1.8% was positive toxoplasmosis using coproscopic examination within 10 years period (2006-2016) in Tirana area. Another factor effect the positive result of toxoplasmosis was age, proven by the research result by Gashout *et al*. [32] that showed at the age of young cats (up to 1-year-old) was 3.22% (6/168), in adults (1-8 years old) was 1.3% (2/153), and at old cats (>9 years old) was 0% (0/121). Furthermore, Nascimentoa *et al*.[33] reported that the PCR assay showed highly sensitive detection results when using <10 tachyzoites of T. gondii DNA and a minimum concentration of 12 ng/ml. The detection limit of the conventional PCR varied depending on the amounts of pure *T. gondii* tachyzoites that were mixed with whole blood. A decreased performance of conventional PCR may be expected when exceeding a certain amount of non-specific DNA in a reaction volume.

Phylogenetic tree construction of *Toxoplasma* (Figure-4) grouped into two clades which clade 1 consists of *T. gondii* repeat region repetitive 529 from India isolate and clade 2 consists of *Toxoplasma* with some strains from GenBank that is *T. gondii* CN DQ779193.1 strain, *T. gondii* NT DQ779188.1 strain, *T. gondii* PYS DQ779189.1 strain, *T. gondii* QHO DQ779190.1 strain, *T. gondii* ZS1 DQ779196.1 strain, and *T. gondii* RH 531 strain (from China) and with some research samples such as toxo control, Yogya pet cat sample T1, T2, and T3 (from Yogyakarta/Indonesia).

The source of infection of *Toxoplasma* in clade 1 was human *Toxoplasma* whereas clade 2 came from animal and human. This research similar to experiment conducted by Franco *et al*. [34] which indicated that Indonesian isolate (IS-1) classified into strain RH, however in this research it has been classified more detail in which IS-1 is grouped into China RH strain which slightly different from clade as India RH strain.

The formation of clade in this research is almost the same as research reported by Hartati [35] in approximately 40 chronic *Toxoplasma* infection animals, and 60 human toxoplasmosis cases showed a high correlation between biological phenotype and genetical characteristic of a certain parasite.

Based on the results, it can be assumed that the sample of toxoplasma used in this study was belonged to strain II that is RH strain and also from animal suffered from a chronic infection. Sibley and Howe [36] stated that there is a difference in replication rate between *T. gondii* Type I, II, and III. *T. gondii* Type I replication rate was higher than Type II and III. Even though statistically it showed not significantly different, the cumulative effect on the amount of cell and tissue destruction showed very significantly different, specifically in the rate of lytic process.

The phylogenetic study had placed the repetitive sequences of RH strain in different clades although the sequence was shared higher homologies among other RH strain from different places [37]. Homology analysis showed that the strain has 90-91% sequence similarity. This is slightly lower than those reported by Christina *et al*. [38], Parmley *et al*. [39], and Costa and Bretagnea [40] which was 92.8%.

## Conclusion

1. Copro-prevalence of toxoplasmosis in pet cats in Yogyakarta by means of PCR, using repetitive 529 bp gene sequences are around 33.33%.

2. The phylogenetic tree of *Toxoplasma* grouped into two clades, which clade 1 consists of *Toxoplasma* isolates collected from pet cats in Yogyakarta Indonesia and *T.gondii* isolates from China and in clade 2 consist of the *T. gondii* isolates from India.

## Authors’ Contributions

MH dan RWN supervised the overall research work. JP, SH, and DA participated in sampling, made available relevant literature and executed the experiment and analyzed the *Toxoplasma* oocysts and DNA sequencing results. All authors interpreted the data, critically revised the manuscript for important intellectual contents and approved the content.
